# The cost-effectiveness of high dose chemotherapy in the treatment of relapsed Hodgkin's disease and non-Hodgkin's lymphoma

**DOI:** 10.1054/bjoc.1999.0880

**Published:** 1999-12-08

**Authors:** S M Beard, P C Lorigan, F C Sampson

**Affiliations:** ScHARR, University of Sheffield, Regent Court, 30 Regent Street, Sheffield, S1 4DA, UK; Department of Clinical Oncology, Weston Park Hospital, Sheffield, S10 2SJ, UK

**Keywords:** Hodgkin's disease, lymphoma, cost-effectiveness, high dose chemotherapy, transplantation

## Abstract

As part of an NHS Executive Trent regional initiative we considered the role and cost-effectiveness of high dose chemotherapy in the treatment of relapsed Hodgkin's disease and non-Hodgkin's lymphoma. The key trials and case series show an additional patient benefit of 0.8–1.1 life years over standard chemotherapy. We estimate incremental cost per life year gained of £12 800–£17 600, which reduces further if long-term benefits are considered. High dose chemotherapy in these conditions is both life-saving and cost-effective. © 2000 Cancer Research Campaign


*British Journal of Cancer* (2000) **82**(1), 81–84
© 2000 Cancer Research Campaign
Article no. bjoc.1999.0880

Short Communication

### The cost-effectiveness of high dose chemotherapy in the treatment of relapsed Hodgkin's disease and non-Hodgkin's lymphoma

**SM Beard[1], PC Lorigan[2] and FC Sampson[1]**

[1]ScHARR, University of Sheffield, Regent Court, 30 Regent Street, Sheffield S1 4DA, UK; [2]Department of Clinical Oncology, Weston Park Hospital, Sheffield S10 2SJ, UK

**Summary** As part of an NHS Executive Trent regional initiative we considered the role and cost-effectiveness of high dose chemotherapy in the treatment of relapsed Hodgkin's disease and non-Hodgkin's lymphoma. The key trials and case series show an additional patient benefit of 0.8–1.1 life years over standard chemotherapy. We estimate incremental cost per life year gained of £12 800–£17 600, which reduces further if long-term benefits are considered. High dose chemotherapy in these conditions is both life-saving and cost-effective. © 2000 Cancer Research Campaign

**Keywords**: Hodgkin's disease; lymphoma; cost-effectiveness; high dose chemotherapy; transplantation

Hodgkin's disease (HD) and non-Hodgkin's lymphoma (NHL) represent approximately 3–5% of all reported malignancies. Whilst the incidence of HD shows a general downwards trend, the overall incidence of NHL is gradually increasing by an estimated 3% per annum. A typical UK health authority (population 500 000) should expect to see between 60 and 70 new cases of NHL and 8–10 new cases of HD each year. Although patients with relapsed disease have historically had a very poor prognosis, new treatment strategies can now cure approximately 40 – 50% of suitable patients.

The use of high dose chemotherapy (HDC) initially supported by autologous bone marrow transplantation (ABMT) and, more recently, peripheral blood stem cells (PBSC), has rapidly become a routine treatment for relapsed lymphoma patients. In studies of relapsed HD patients, long-term survival rates of 50–65% have been reported using various HDC regimens (Reece et al, 1991; Bierman et al, 1993; Chopra et al, 1993; Goldstone et al, 1993). Similar results have been shown for relapsed high/intermediate grade NHL patients (Philip et al, 1991, 1995; Salzman et al, 1997). In a recent review (Beard et al, 1998) we considered the role and cost-effectiveness of HDC in the treatment of lymphoma for the Trent Institute Working Group on Acute Purchasing (TIWGAP), a regional body established to consider evidence of clinical and cost-effectiveness for new drugs and interventions. We present our pharmacoeconomic findings, and draw on the key messages of cost-effectiveness.

### METHODS

We focused specifically on patients with relapsed HD and relapsed high/intermediate grade NHL, as these are the areas where clinical evidence of efficacy is most convincing.

*Received 14 December 1998*
*Revised 21 June 1999*
*Accepted 8 July 1999*

*Correspondence to:* SM Beard

A systematic literature search of Medline, Embase and BIDS Science Citation Index identifying all trials of HDC in lymphoma was carried out, to include all trials up until end January 1998. Results were cross-referenced against the references used within the ongoing HTA Systematic Review (now published – Johnson et al, 1998). Although randomized control trials (RCTs) were targeted as a 'gold standard', there is very little RCT evidence available in this area and evidence from large peer-reviewed case series was also considered. As curative therapy was the basis of HDC treatment the primary outcome measure of this analysis was cost per life year gained (LYG). In estimating the survival benefits of HDC against those of standard salvage chemotherapy, we adopted a methodology based on area under the curve (AUC) estimation, where the AUC was taken directly from the published Kaplan–Meier graphs. This approach allows the experience of the whole trial arm to be acknowledged rather than using a single point estimate, such as the median survival statistic.

As trial data suggest a longer term survival for HDC we included a consideration of potential longer term benefits, i.e. those lying beyond the trial period, based on an extrapolation of published survival data. We adopted a conservative approach in terms of interpretation of clinical benefits and inclusion of treatment costs.

Guidelines for costing chemotherapy have been issued by the National Health Service Executive (NHS Management Executive, 1993). Briefly, these costs are split into fixed, semi-fixed and variable costs and include a portion of all costs incurred in running a hospital, on a pro-rata basis. The costs of standard chemotherapy, salvage chemotherapy and high dose therapy at this institution have previously been estimated (Hancock et al, 1995). However, these costs are unique to this unit and depend on specific case mix, activity, specialist facilities etc. Whilst they are very useful for the purpose of contracting, they are not easily transferable to another unit. A more durable assessment of cost can be gained simply from drug acquisition costs and time in hospital. This can then be supplemented with specific costs applicable to that unit. The Out of Area Treatment (OATS) cost for high dose therapy at this unit is

**Table 1** High dose chemotherapy survival benefits and cost per LYG

| Forward Projection of Trial Data | Hodgkin's disease BNLI Trial | | | Non-Hodgkin's lymphoma PARMA Trial | | |
|---|---|---|---|---|---|---|
| | Survival Benefit (mths) | LYG | Cost per LYG | Survival benefit (mths) | LYG | Cost per LYG |
| Initial trial data only | 10 | 0.8 | £17 375 | 13 | 1.1 | £12 636 |
| 5 years extended | 28 | 2.3 | £6 043 | 27 | 2.3 | £6043 |
| 10 years extended | 45 | 3.8 | £3 658 | 40 | 3.3 | £4212 |
| 20 years extended | 78 | 6.5 | £2 138 | 66 | 5.5 | £2527 |
| Long-term trial follow-up data (2 years extended) | n/a | n/a | n/a | 18 | 1.5 | £9267 |

£15 500. The acquisition cost for standard first line CHOP chemotherapy is £960, and the total cost rises to £1700 if one assumes a 50% chance of neutropenic sepsis. This cost includes 5 days of antibiotics, 2 unit blood transfusion, 5 units of platelets, antifungal therapy etc. We have based our cost for standard dose salvage therapy on these costs although salvage treatment for NHL tends to be individualized and is likely to be significantly more expensive than standard first-line CHOP chemotherapy. The mean cost of palliative therapy for Hodgkin's disease, including time spent in hospital, antibiotic usage etc. has been calculated at £9500 for this unit. The costs above do not include the costs borne by palliative care, primary care etc.

It is likely that the costs for standard salvage therapy are an underestimate, with the result that high dose chemotherapy will look disproportionately more expensive when compared to standard therapy. The marginal cost of HDC is estimated to be approximately £13 900, based on first-line therapy alone.

A sensitivity analysis was conducted in order to explore the potential influence of variance in both treatment costs and benefits. We allowed the marginal cost of HDC to vary between £10 000 and £20 000 to consider the influence of including salvage therapy costs and the potential regional variations in HDC costs. We also explored a ± 50% variation around the marginal clinical benefits of HDC.

### RESULTS

### Marginal benefit of HDC in relapsed HD patients

The British National Lymphoma Investigation (Linch et al, 1993) showed improved overall response rates for BEAM+ABMT versus mini-BEAM (74% vs 60%, $P > 0.1$) and increased 3 years actuarial event-free survival (53% vs 10%, $P < 0.05$). Overall, 15 patients remained alive after 34 months follow-up, compared to 11 in mini-BEAM arm ($P = 0.318$). This trial failed to reach the recruitment target as patients were refusing randomization in favour of HDC.

HDC was estimated to provide a marginal survival benefit of 10 months per patient, with the plateau of the survival curve indicating long-term survival with likely prolonged benefits beyond the trial end point. Marginal survival benefits and corresponding cost per LYG figures, for trial data alone and time periods at 5, 10, and 20 years beyond the end of the trial data are shown in Table 1. The majority of the impact is apparent in the first 5 years beyond the trial end point.

In addition, we compared data from the larger HDC case series (Chopra et al, 1993) with outcomes of conventionally treated HD patients, as suggested by the National Cancer institute (NCI) series (Longo et al, 1992). This provided a similar magnitude of benefit for HDC, with marginal survival benefit at 11 months. A recent randomized study from the EBMT comparing standard therapy with HDC in relapsed HD which closed early due to poor accrual, showed no survival advantage for the HDC arm (Schmitz et al, 1999). However, this was because the majority of patients who relapsed after standard therapy were then salvaged with HDC. Full details are not yet available but the authors concluded that HDC is still the treatment of choice for HD in first relapse.

### Marginal benefits of HDC in relapsed NHL patients

The PARMA group (Philip et al, 1995) showed an initial response rate of 84% after HDC and 44% after standard salvage treatment in relapsed NHL patients. At 5 years the event-free survival was significantly greater in the HDC arm (46% vs 12%, $P = 0.001$) and overall survival was also superior (53% vs 32%). The marginal survival benefit was calculated as 13 months (1.1 LYG) per patient, in favour of HDC, and again a forward projection of benefits was taken beyond the original trial period (Table 1). Recently updated trial data (Philip et al, 1998), taken over an 8-year follow-up period, suggests that patient survival benefits are sustained and significant differences remain over the long-term. This provides further support to our original projection of benefits beyond the initial trial period. The 8-year event-free survival (36% vs 11%, $P < 0.002$) and overall survival (47% vs 27%, $P < 0.042$) figures correspond to an estimated marginal survival benefit of 18 months (1.5 LYG) and a cost per LYG of £9267 (Table 1).

### Sensitivity analysis

The results of the sensitivity analysis are summarized in graphical form, allowing different combinations of costs and benefits to be considered as required (Figures 1 and 2). Each graph shows three separate series, corresponding to different levels of clinical benefit and representing a level of cost-effectiveness, tracking cost per life year values against the marginal cost of HDC. If the benefits are assumed to be equal to those seen within the trial the cost-effectiveness of HDC in both NHL and HD remains under £25 000 per LYG, even when the marginal cost of HDC is increased to £20 000.

 © 2000 Cancer Research Campaign

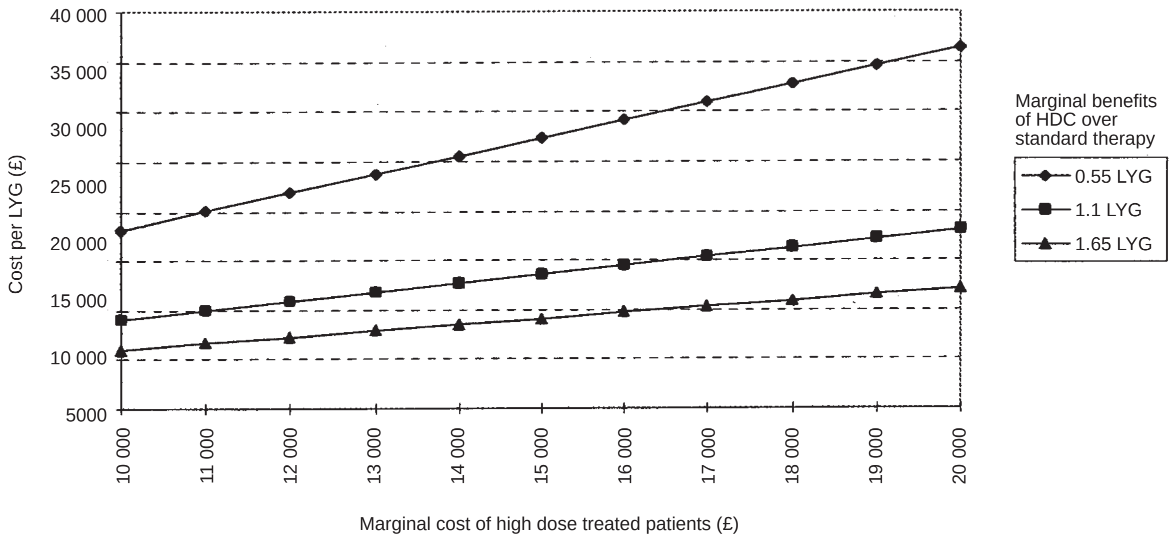

**Figure 1** Cost-effectiveness sensitivity in NHL patients

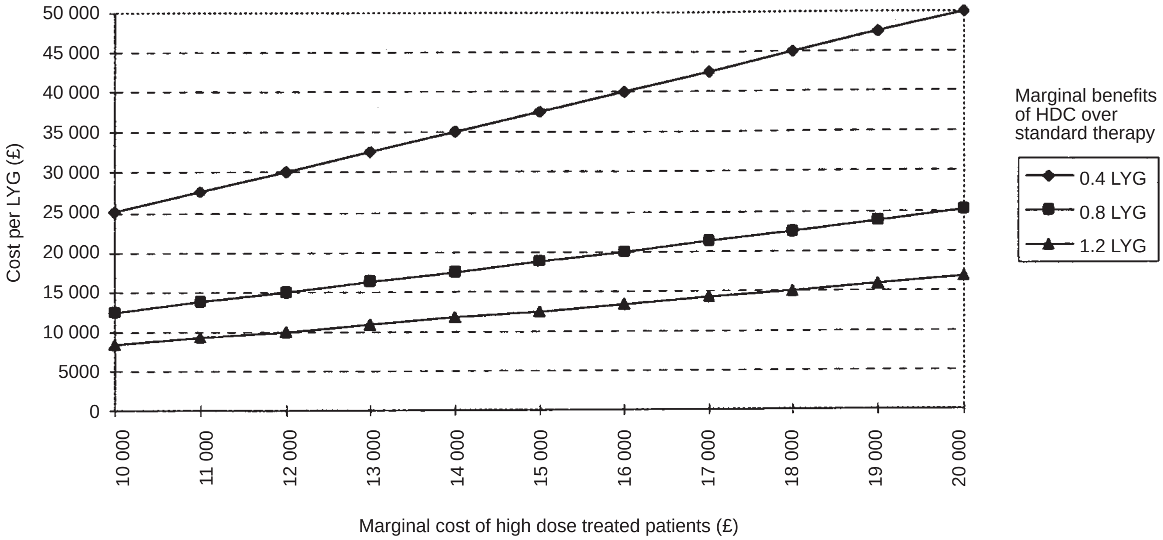

**Figure 2** Cost-effectiveness sensitivity in HD patients

### DISCUSSION

The strength of observational study evidence strongly supports the use of HDC in relapsed HD and relapsed high/intermediate grade NHL. There is little RCT evidence available and further trials are unlikely to take place due to recruitment difficulties for the standard therapy arm. The BNLI trial did not reach a statistically significant survival advantage as the trial was stopped early. The EBMT trial was essentially a study comparing early with late HDC in relapsed Hodgkin's disease. Recently revised EBMT guidelines provide a useful framework within which to consider the role of HDC for treatment of HD and NHL (Goldman et al, 1998).

The cost-effectiveness arguments for the use of HDC in relapsed NHL and relapsed HD patients are strong, even under sensitivity analysis involving both costs and benefits. The cost-effectiveness ratios, at £12 636 and £17 375 respectively, are certainly comparable with similarly supported therapies and fall under generally accepted UK cost-effectiveness thresholds of around £20 000 per LYG (Stevens et al, 1995). Even using these conservative cost estimates, if 5-year projected benefits are included the cost per LYG figures are reduced by around 50%.

The actual marginal cost of HDC may be significantly lower than that used in our analysis as the high initial costs of HDC would be partially offset by the reduced likelihood of further chemotherapy following relapse. Patients with high grade NHL who relapse tend to have aggressive disease and do not survive very long. Patients with relapsed HD may exhibit a chronic relapsing remitting condition requiring regular treatment and supportive care, but long-term survival is unusual. Furthermore, this analysis has not taken into account difference in the quality of life between patients cured of their lymphoma and those receiving palliative therapy for incurable disease.

Cost-effectiveness analyses of new treatments are an integral part of the move towards evidence-based purchasing for all health services, including oncology. The establishment of the National Institute for Clinical Excellence (NICE) has formalized the efforts

*© 2000 Cancer Research Campaign*

of TIWGAP and similar regional development and evaluation bodies currently conducting cost-effectiveness analysis for the Inter-DEC (Department of Health, 1998). Clinicians and purchasers alike need to remain aware of cost-effectiveness issues and empower themselves in such an evidence-based environment.

High dose chemotherapy in relapsed HD and NHL is both a clinically effective and cost-effective treatment strategy.

### REFERENCES

Beard SM, Lorigan P, Sampson F and Sims A (1998) The effectiveness of high dose chemotherapy with autologous stem cell transplantation in the treatment of Hodgkin's disease and non-Hodgkin's lymphoma. *Sheffield: Trent Institute for Health Services Research, Universities of Leicester, Nottingham and Sheffield. Guidance Note for Purchasers*: 98/04

Bierman PJ, Bagin RG, Jagannath S, et al (1993) High-dose chemotherapy followed by autologous hematopoietic rescue in Hodgkin's disease: long-term follow-up in 128 patients. *Ann Oncol* **4**: 767–773

Chopra R, McMillan AR, Linch DC, et al (1993) The place of high dose BEAM therapy with autologous bone marrow transplantation in poor risk Hodgkin's disease: a single centre 8-year study of 155 patients. *Blood* **81**: 1137–1145

Department of Health (1998) *A first class service – quality in the new NHS*. Department of Health: London

Goldman JM, Schmits N, Niethammer D and Gratwohl A (1998) Allogeneic and autologous transplantation for haematological diseases, solid tumours and immune disorders: current practice in Europe in 1998. *Bone Marrow Transpl* **21**: 1–7

Goldstone AH and McMillan AK (1993) The place of high-dose therapy with haemopoietic stem cell transplantation in relapsed and refractory Hodgkin's disease. *Ann Oncol* supp 1: S21–S27

Hancock BW and Barber A (1995) The cost effectiveness of cancer chemotherapy: a clinicians view. *Proc R Coll Physic Edinburgh* **25**: 61–66

Johnson PWM, Simnett SJ, Sweetenham JW et al (1998) Bone marrow and peripheral blood stem cell transplantation for malignancy. *Health Technol Assess* **2**: 8

Linch DC, Winfield D, Goldstone AH, et al (1993) Dose intensification with autologous bone marrow transplantation in relapsed and resistant Hodgkin's disease: results of a BNLI randomised trial. *Lancet* **34**: 1051–1054

Longo DL, Duffy PL, Young RC, et al (1992) Conventional dose salvage combination chemotherapy in patients relapsing with Hodgkin's disease after combination chemotherapy: the low probability of cure. *J Clin Oncol* **10**: 210–218

NHS Management Executive (1993) *Costs for Contracting*. FDL (93) 59 Department of Health.

Philip T, Chauvin F, Armitage J, Bran D, et al (1991) PARMA International Protocol: pilot study of DHAP followed by involved field radiotherapy and high dose chemotherapy with autologous bone marrow transplantation. *Blood* **77**: 1587–1592

Philip T, Gulglielmi C, Hagenbeek A, et al (1995) Autologous bone marrow transplantation as compared with salvage chemotherapy in relapses of chemosensitive non-Hodgkin's lymphoma. *N Engl J Med* **33**: 1540–1545

Philip T, Gomez F, Guglielmi G et al (1998) Long-term outcome of relapsed non-Hodgkin's lymphoma (NHL) patients included in the PARMA trial: incidence of late relapses, long-term toxicity and impact of the international prognostic index (IP) at relapse. *Proc Am Soc Clin Oncol* **17**: A62

Reece DE, Barnett MJ, Connors JM, et al (1991) Intensive chemotherapy with cyclophosphamide, carmustine and etoposide followed by autologous bone marrow transplantation for relapsed Hodgkin's disease. *J Clin Oncol* **9**: 1871–1879

Salzman DE, Briggs AD and Vaughan WP (1997) Bone marrow transplantation for non-Hodgkin's lymphoma: a review. *Am J Med Sci* **313**: 228–235

Schmitz N, Sextro M, Pfistner B, et al (1999) High dose therapy followed by haematopoietic stem cell transplantation for relapsed chemosensitive Hodgkin's disease: final results of a randomised GHSG and EBMT trial (HD-R1). Available from internet at: http://asco/infostreet.com/prof/httml/99abstracts/all/m_5.htm

Stevens A, Colin-Jones D and Gabbay J (1995) 'Quick and clean': authoritative health technology assessment for local health care contracting. *Health Trends* **27**: 37–42

Weisdorf DJ, Anderson JW, Glick JH, et al (1992) Survival after relapse of low grade non-Hodgkin's lymphoma: implications for marrow transplantation. *J Clin Oncol* **10**: 942–947

*© 2000 Cancer Research Campaign*

